# Ultrasound-guided percutaneous renal biopsy-induced accessory renal artery bleeding in an amyloidosis patient

**DOI:** 10.1186/1746-1596-7-176

**Published:** 2012-12-11

**Authors:** Qing Zhang, Yongqiang Ji, Tianwei He, Jianping Wang

**Affiliations:** 1 Department of Nephrology, Yantai Yuhuangding Hospital, Qingdao University Medical College, Yantai 264000, China

**Keywords:** Kidney, Biopsy, Amyloidosis, Accessory renal artery, Hemorrhage

## Abstract

**Abstract:**

Ultrasound-guided percutaneous renal biopsy is an important technique for diagnosis of glomerular diseases, and the biopsy-induced life-threatening bleeding rarely happens. Primary systemic amyloidosis is a rare disease which may lead to organ dysfunction including arterial stiffness. The accessory renal artery is a kind of renal vascular variation which goes into the renal parenchyma directly or via the renal hilum. Here we reported a rare case of percutaneous renal biopsy-induced accessory renal artery life-threatening bleeding in a renal amyloidosis patient, and our experience of successful rescue in this patient.

**Virtual Slides:**

http://www.diagnosticpathology.diagnomx.eu/vs/1524207344817819

## Introduction

Ultrasound-guided percutaneous renal biopsy is an important technique for diagnosis and providing evidence for the treatment plans for glomerular diseases. However, percutaneous renal biopsy may also have some side effects and kidney damage such as pain at the puncture site, microscopic or gross hematuria, and perirenal hematoma [1]. Usually these side effects can be alleviated after bed rest and treatment of hemostasis, and life-threatening bleeding rarely happens. Renal amyloidosis is a rare disease accounting for 0.21%-1.0% of all the patients with renal biopsy. Primary systemic (AL) amyloidosis is a clonal plasma cell disorder in which the N-terminal fragments of monoclonal light chains form fibrils that accumulate in various organs ultimately leading to organ dysfunction and death [2]. This disease is difficult to recognize and differentiate from other renal diseases [3-5] with poor prognosis because of its broad range of manifestations and vague symptoms. The accessory renal artery is a kind of renal vascular variation, which has been seen in 28-30% of normal subjects. It goes into the renal parenchyma directly or via the renal hilum, and happens more in the left than the right kidney [6]. Here we reported a rare case of percutaneous renal biopsy-induced accessory renal artery bleeding in a renal amyloidosis patient, and our experience of successful rescue in this patient.

## Case presentation

A 67-year old male was found having abnormalities in urine tests (blood -, protein ++) during treatment for his “mixed hemorrhoids” in the inpatient unit two months ago. But these abnormalities were not further investigated at that time. 20 days ago, the results of urine tests were: blood ++; protein +++; albumin 32.9 g/L. Eight days ago, the results were: blood +, protein +++; albumin 28.94 g/L, uric acid 568 umol/L. Patient did not have any of the following symptoms: fever, joint pain, hair loss, mouth ulcers, rash, gross hematuria, urinary discomfort or dysuria, skin purpura, abdominal pain, melena, headache, dizziness, chest tightness, suffocation, palpitation, lumbar pain, or other symptoms. The patient has had hypertension and token antihypertensive drugs for 10 years. But in the past six months, his blood pressure was normal and he stopped antihypertensive drugs. He had repeated diarrhea with body weight loss of about 25 kg in the past six months. His father passed away 10 years ago due to “uremia”, and he denied any familial genetic history. The diagnosis was “nephrotic syndrome”.

Physical examination when hospitalized did not find anything abnormal in his lungs, heart, abdomen, and limbs. There was no bleeding or ecchymosis of the skin. Body temperature 36.4°C, pulse 82 beats/min, respiratory 20 beats/min, blood pressure 108/68 mmHg. Urine test: protein ++, blood -. Fecal occult blood test: negative. Blood test: WBC (white blood cell) 4.9 × 10^9^/L, HB 130 g/L, platelet 122 × 10^9^/L, albumin 29.57 g/L, BUN (blood urea nitrogen) 6.0 mmol/L, serum creatinine 98 μmol/L, uric acid 490.7 μmol/L, total cholesterol 5.2 mmol/L, triglycerides 2.89 mmol/L. Humoral immune function: complement C3: 0.876 g/L (slightly lower); urine total protein 5.30 g/24 h; The results from all the following tests were normal: erythrocyte sedimentation rate, coagulation, urine red blood cell morphology, hepatitis B, autoantibodies, antineutrophil cytoplasmic antibodies (ANCA), proteinase-3 (PR3), MPO (myeloperoxidase), urine and blood light chain. Echocardiography showed left ventricular dilatation and diastolic dysfunction. Other tests showed the hyperactivity of bone marrow cell proliferation, 3% of mature plasma cells.

**Figure 1:**
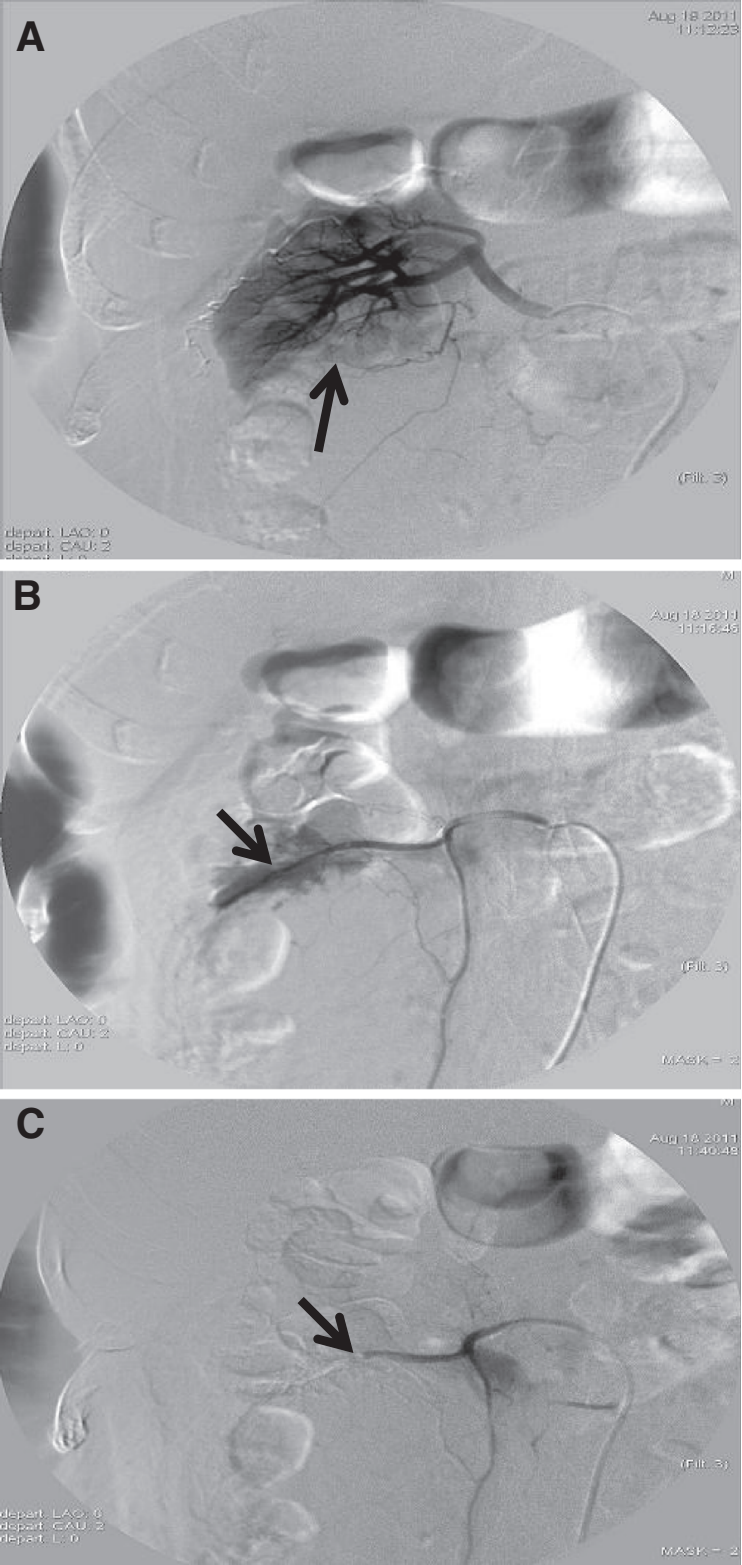
**Images from right renal arteriography.** (**A**) The arrow points to the lower pole of right kidney without renal blood flow. (**B**) The arrow points to an accessory renal artery from the abdominal aorta with bleeding in the lower pole of right kidney. (**C**) The right accessory renal arterial bleeding stopped after the vascular embolization with gelatin sponge particles.

To provide further evidence for the diagnosis and treatment plans, we performed ultrasound-guided percutaneous renal biopsy at the lower pole of right kidney. At half an hour after the biopsy, the patient had nausea, vomiting, abdominal pain, sweating, and discomfort on the right side of the waist. Blood pressure decreased to 90/50 mmHg. Hemoglobin was 110 g/L. Ultrasonography showed a 4.5 cm × 6.0 cm of hematoma in the lower pole of right kidney. After treatments with hemostasis, blood transfusion and the pressor agent, symptoms were improved and blood pressure rose up to 98/70 mmHg in four hours. 24 hours later, he walked around his bed and had abdominal pain again with sweating. Blood pressure decreased, hemoglobin dropped to 78 g/L. Abdominal computed tomography (CT) showed hematoma in perinephric and retroperitoneal area. Renal artery angiography found severe bleeding from the accessory renal artery at the lower pole of the right kidney (Figure 1B). After the application of the accessory renal artery embolization with gelatin sponge particles, bleeding was stopped and blood pressure increased to normal in ten minutes (Figure 1C). Hemoglobin returned to normal in 10 days after the surgery.

Microscopy with immunostaining showed positive staining of IgG + and IgM + in the glomerular mesangial areas and capillary loops. Clumped distribution of light chain κ++ and λ + were seen in the mesangial area, capillary loops, interstitial vascular wall and parts of the tubular basement membrane. AA protein (amyloid protein A) was negative.

**Figure 2:**
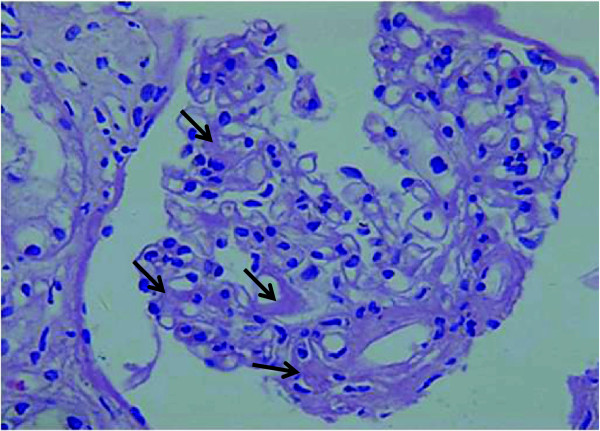
**Hematoxylin and eosin staining (X400).** The arrows point to lightly stained homogeneous structure in glomerular mesangial areas.

**Figure 3:**
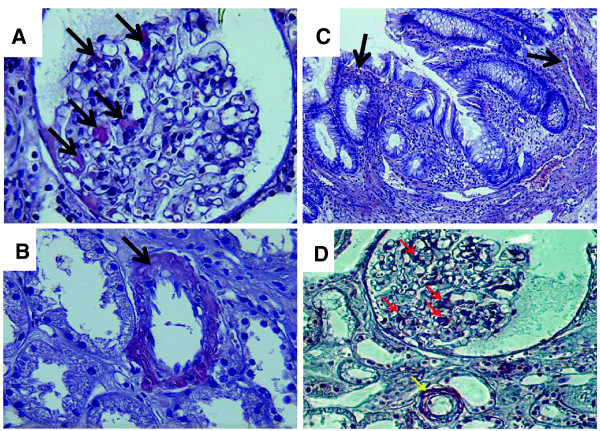
**Congo red staining (X400).** The arrows point to Congo red positive material deposition in glomerular mesangial areas (**A**), walls of the renal arteries (**B**), and the intestine (**C**). (**D**) A polarization image of the Congo red stain shows positive material deposition in glomerular mesangial areas (red arrows) and walls of the renal arteries (yellow arrows).

Light microscopy showed three out of 16 glomeruli with global sclerosis, and mesangial cells and matrix mildly and diffusely proliferated in other glomeruli. Unstructured homogeneous nodules with mild pale staining have been seen in the mesangial area and the basement membrane. The capillary lumen was poorly open. Bowman’s capsule wall was segmentally thickening and its epithelial cells proliferated with adhesion to Bowman’s capsule. PASM-MASSON staining showed that the glomerular basement membrane was segmental thickening with tubular atrophy. The tubular wall without atrophy was mild thickening. Vacuolation and granular degeneration appeared in the tubular epithelial cells. Some of the tubular epithelial cells became flat, with expanded lumen, protein cast in the lumen, and brush border’s defluvium. Other pathological findings: focal tubulointerstitial fibrosis (++), inner layer of the interstitial arterial wall thickening, and deposited amorphous materials with mild staining in middle and outer layers (Figure 2). Congo Red staining showed deposition of uniform brick red material in glomerular mesangial and tubulointerstitial regions, the basement membrane, and renal interstitium vascular wall (Figure 3A-B). Congo Red staining in intestinal tissue was positive (Figure 3C).

Electron microscopic examination revealed mild increase of segmental space without visible cells in the glomerular mesangial area, visible clutter of amyloid fibrils in the cavity, segmental fusion of the foot processes of podocytes, increased lysosome in renal tubular epithelium, renal interstitial few lymphocytes and mononuclear cells infiltration with collagen fiber hyperplasia.

## Discussion

Primary systemic amyloidosis is a malignant plasma cell disease. Classification of the amyloidosis is based on the precursor protein that forms the amyloid fibrils and the distribution of amyloid deposition as either systemic or localized. To date, 25 structurally unrelated proteins are known to cause amyloidosis [7]. The major types of systemic amyloidosis are Ig light chain (AL), Ig heavy chain (AH), amyloid A (AA), the familial or hereditary amyloidosis, senile systemic amyloidosis, and β2-microglobulin (β2m) amyloidosis. In AL amyloidosis, an immunoglobulin (Ig) light chain or light chain fragment produced by clonal plasma cells deposits in tissue as amyloid [7]. In this case, the immunohistochemistry study showed renal IgG+, IgG light chain κ++ and λ+, suggesting it was AL amyloidosis in this case. The incidence of AL amyloidosis in the United States is estimated to be between 5.1 and 12.8 per million persons per year [8]. The kidney is affected in 50 to 80% of AL amyloid individuals [9-12]. The spectrum of renal symptoms and signs in amyloidosis is variable such as isolated proteinuria, nephrotic syndrome, hypertension, hypotension, renal insufficiency [13]. Compared with other glomerular diseases, the kidney disease with primary systemic amyloidosis has poor prognosis, gets progressively worse over time and the kidney lesions are irreversible. The amyloidosis can also induce the damages in the heart, autonomic nervous system, gastrointestinal tract and other tissues, resulting in arrhythmia, heart failure, refractory orthostatic hypotension, severe diarrhea and other organ dysfunctions. Patient’s overall conditions are usually poor. It was reported that the median survival time was only 1.2 years [10,14]. Early diagnosis and treatment is critical for the prognosis of this disease. Renal involvement is diagnosed through the evidence of renal amyloid deposits detected through biopsy, together with laboratory evidence of kidney dysfunction. Positive Congo red staining in the lesion area and the specific apple green birefringence under polarized fluorescence microscope provide evidence for the diagnosis of this disease. Combined with blood and urine electrophoresis analysis for immunoglobulin free light chain or the quantitative analysis of serum free light chain which confirms the presence of monoclonal free light chain, the sensitivity of diagnosis of systemic light chain amyloidosis is up to 99% [15]. The treatment principles and prognosis are different among different types of renal amyloidosis. Reducing the production of amyloidogenic precursor protein (AA and AL amyloidosis) and enhancing the clearance of amyloidogenic precursor protein (Aβ2M amyloidosis) as well as trying to break down the amyloid deposits are the aims of the therapy. Once end-stage renal disease develops, patients can be treated with either dialysis or renal transplantation. In patients with adequate criteria, stem cell transplantation has shown encouraging results in several recent studies, and the five-year survival rate has been estimated to be approximately 60% [16,17].

The old male patient in this report had repeated diarrhea for six months and resisted to a variety of drug treatment. Colonoscopy showed nothing abnormal. He had the history of hypertension, but in the past six months his blood pressure was normal and he did not take any antihypertensive drugs. Lab tests found urine protein and decreased serum albumin, without evidence of myeloma. Renal biopsy and histological study showed the deposition of homogeneous material in the glomerular mesangial area and interstitial vessel wall (Figure 2), and positive Congo red staining (Figure 3). The immunofluorescence staining showed light chain κ++, λ+, pervaded in the mesangium, capillary loops, interstitial vessel wall and part of the tubular basement membrane. Amyloid protein A (−) confirmed for the AL-type renal amyloidosis. Electron microscopy showed mild segmental mesangial widened, amyloid fibers cluttered in the cavity, which further confirmed the diagnosis of renal AL-type amyloidosis. It also confirmed that amyloidosis not only affected glomeruluser, but also the interstitial blood vessels. Due to the tendency of the amyloid deposition in small blood vessels, the bleeding risk induced by kidney biopsy is increased. So the 18X16G fine puncture needle was used in the process of puncture. In this patient, there was more amyloid deposition in the vessel walls, which might be the main reason for the difficulty to stop bleeding. The reason for the repeated diarrhea in this patient was not clear. However, the intestinal amyloid deposition has been found by the Congo red staining in colonic tissue (Figure 3). As the systemic symptoms improved after the subsequent treatment, the diarrhea had stopped completely.

The anatomical variations of renal vasculation are common and it is easy to be found in Urologic Surgery. But because the renal arteriography is not routinely performed before the kidney biopsy in the department of nephrology, the situation of accessory renal artery is difficult to be assessed. The incidence of accessory renal artery in left kidney is higher than that in the right kidney [18]. The diseases related to the renal accessory artery include: (1) stenosis: renal accessory artery is tenuous and tortuous, which results in ischemia in the related area. The local blood flow reduction in the kidney stimulates the macula densa cells and juxtaglomerular cells to increase the synthesis and release of renin, and the subsequent renal vascular hypertension. (2) bleeding: non-traumatic renal bleeding is very rare. As the accessory renal artery is tenuous, it is difficult for the diagnosis and the localization for the hemorrhage. New techniques, such as multidetector CT angiography (MDCTA) which can clearly show the accessory renal arteries, provide better approaches for the accessory renal artery disease. (3) Accessory renal artery going to the lower pole of the kidney in front of ureter may compress the ureter and cause hydronephrosis. In this patient, we did renal biopsy from the lower pole of the right kidney and unfortunately hurt the accessory renal artery resulting in bleeding (Figure 1). There has been seldom damage to the renal artery during the puncture because a healthy artery is contractive with hemostatic function when a fine needle hits it. But in this patient of amyloidosis with the amyloidosis deposition mainly in blood vessel walls, the puncture caused serious bleeding. The conservative treatments were difficult to stop the bleeding. So the renal arteriography and the vascular embolization with gelatin sponge particles has been finally used (Figure 1). We have learned from this case that once large amount of bleeding occurred during renal biopsy for the patient with renal amyloidosis, renal arteriography and vascular embolization should be performed immediately to avoid adverse consequences.

## Consent

Written informed consent was obtained from the patient for publication of this case report and any accompanying images.

## Competing interests

The authors declare that they have no competing interests.

## Authors’ contributions

QZ conceived the study and wrote the manuscript. YJ, TH, and JW performed the microscopic sections and analysis. All authors read and approved the final manuscript.
